# Admission Cardiotocography in Term Pregnancies for Predicting Intrapartum Interventions and Early Neonatal Outcomes: A Systematic Review

**DOI:** 10.7759/cureus.109070

**Published:** 2026-05-18

**Authors:** Shamili Priya Jha, Swati Kumari, Aprajita Sinha, Rajib Roy, Sanjita, Suman Kumari, Lata

**Affiliations:** 1 Department of Obstetrics and Gynaecology, Employees State Insurance Corporation (ESIC) Medical College and Hospital, Bihta, Patna, IND

**Keywords:** admission cardiotocography, fetal monitoring, intrapartum outcomes, neonatal outcomes, term pregnancy

## Abstract

Cardiotocography (CTG) is widely applied for intrapartum fetal surveillance to identify early signs of fetal compromise and guide timely intervention. Despite extensive clinical use, uncertainty persists regarding its effectiveness in improving neonatal outcomes, particularly when used routinely at admission in low-risk term pregnancies. This systematic review addresses inconsistencies in existing evidence concerning the predictive value and clinical utility of admission CTG. The objective was to evaluate its association with intrapartum interventions and early neonatal outcomes. A systematic review design was employed, conducted in accordance with Preferred Reporting Items for Systematic Reviews and Meta-Analyses (PRISMA) 2020 reporting principles where applicable, analysing studies published between 2000 and 2026 across databases including PubMed, Scopus, Cochrane Library, and Web of Science. No prospective protocol registration was undertaken. Eligible studies included randomized controlled trials and cohort, cross-sectional, and observational designs focusing on term pregnancies. Following screening and eligibility assessment, nine primary studies were included in the qualitative synthesis. Data were extracted and synthesized qualitatively due to clinical and methodological heterogeneity, including differences in study design, population risk status, admission CTG classification criteria, sample size, and outcome definitions. Risk-of-bias assessment showed that most included studies had a moderate overall risk of bias, mainly due to selection bias, lack of blinding, variability in CTG interpretation, and inconsistent outcome assessment. The findings indicate that abnormal admission CTG patterns are associated with increased rates of cesarean section, instrumental delivery, low Apgar scores, and higher neonatal intensive care unit (NICU) admissions, while demonstrating variable diagnostic performance. The reported sensitivity and specificity for predicting low Apgar scores were 66.7% and 93.3%, respectively, whereas other outcomes showed poorer sensitivity and positive predictive value despite higher specificity and negative predictive value. Clinical implications highlight the potential for over-intervention influenced by clinical interpretation without proportional neonatal benefit. Admission CTG remains valuable as a screening tool when applied selectively based on risk stratification. The evidence supports cautious interpretation integrated with clinical assessment. Overall, the findings support selective, risk-based use of admission CTG rather than routine implementation in all low-risk term pregnancies.

## Introduction and background

Cardiotocography (CTG) has become one of the main tools in modern obstetrics to assess fetal well-being during labor by monitoring fetal heart rate patterns and uterine contractions [1]. It is used clinically on the premise that timely detection of fetal hypoxia may facilitate appropriate intervention and reduce perinatal morbidity and mortality [2]. CTG may be used either as continuous intrapartum monitoring throughout labor or as a short admission test at the time of entry to the labor ward [3]. These two approaches differ in purpose: continuous intrapartum CTG is used for ongoing surveillance during labor, whereas admission CTG is intended as an initial screening assessment to identify fetuses that may require closer monitoring or intervention. Admission CTG refers to a short fetal heart rate and uterine contraction recording, usually lasting approximately 20 minutes, performed when a pregnant woman is admitted to the labor ward before or during the early phase of labor [4]. Its purpose is to classify the fetal heart rate pattern as reassuring/reactive, suspicious/non-reassuring, or pathological, and to guide subsequent intrapartum monitoring and management.

Term pregnancies are clinically important because fetuses at ≥37 weeks of gestation are generally expected to tolerate labor, yet acute intrapartum events such as cord compression, uteroplacental insufficiency, meconium-stained liquor, or evolving hypoxia may still occur despite the absence of antenatal complications [5]. In this population, the balance between detecting unexpected fetal compromise and avoiding unnecessary obstetric intervention is especially important because false-positive or equivocal CTG findings may increase cesarean sections or instrumental delivery without a corresponding neonatal benefit [6]. This balance is central to the clinical debate surrounding routine admission CTG, particularly in low-risk term pregnancies.

Admission CTG has been proposed as a screening tool to identify fetuses at risk before active labor or before major labor-management decisions are made [7]. Although some studies suggest that admission CTG facilitates early recognition of fetal compromise, evidence regarding its effect on neonatal outcomes remains inconsistent [8]. The controversy is most pronounced in low-risk term pregnancies, where routine admission CTG may convert a clinically uncomplicated labor into a higher-intervention pathway due to suspicious or non-reassuring tracings with limited positive predictive value. In such cases, abnormal CTG findings may reflect transient physiological variation rather than clinically significant fetal compromise, creating uncertainty regarding whether routine screening improves outcomes or mainly increases operative delivery [9].

Adjunctive techniques, such as fetal electrocardiogram (ECG) ST-segment waveform analysis, have been proposed to enhance diagnostic accuracy; however, their impact on clinical outcomes remains inconsistent across different populations and healthcare settings [5]. Because these adjunctive and automated approaches are not yet consistently validated for routine admission CTG assessment, they are discussed only as contextual developments rather than as core evidence for this review [10]. Current clinical decision-making, therefore, still relies largely on conventional CTG interpretation integrated with maternal and fetal risk assessment.

Variability in CTG interpretation remains a major limitation. Substantial interobserver and intraobserver differences have been reported, with only moderate agreement among clinicians interpreting CTG tracings [10]. This subjectivity affects clinical decision-making and limits the reliability of CTG as a standalone diagnostic tool. Intermittent auscultation has demonstrated comparable neonatal outcomes with fewer obstetric interventions in low-risk pregnancies, raising concerns regarding routine CTG use in such populations [11]. Standardized interpretation frameworks and integration with clinical assessment are therefore essential when admission CTG is used as a screening tool.

Prior reviews have provided valuable evidence on intrapartum fetal monitoring, but several limitations remain. Some reviews have evaluated continuous CTG during labor rather than admission CTG specifically, while others have combined low-risk and high-risk pregnancies, included non-term populations, or focused mainly on intervention rates without adequately characterizing diagnostic accuracy, early neonatal outcomes, and risk-of-bias patterns [12]. In addition, heterogeneity in CTG classification systems, outcome definitions, and clinical management protocols has limited the direct applicability of earlier findings to routine admission assessment in term pregnancies. This review, therefore, prioritized literature directly related to admission CTG, intrapartum fetal surveillance, CTG interpretation, diagnostic performance, and early neonatal outcomes [13].

The specific knowledge gap addressed by this review is whether admission CTG has sufficient predictive utility to identify term pregnancies at risk of intrapartum compromise and early adverse neonatal outcomes without increasing unnecessary obstetric intervention. This gap is clinically relevant because admission CTG may increase intervention rates even when neonatal benefit is uncertain [14]. Admission CTG is commonly associated with increased obstetric interventions, including cesarean section and instrumental delivery [15]. While abnormal CTG patterns are associated with adverse neonatal outcomes, such as low Apgar scores and increased neonatal intensive care unit (NICU) admissions, the predictive value remains inconsistent, reflecting variability in sensitivity and specificity across studies as well as the influence of clinical interpretation on management decisions [16].

Current evidence supports a selective, risk-based approach to CTG use rather than routine implementation in low-risk pregnancies [17]. This review therefore focuses specifically on admission CTG in term pregnancies, with emphasis on its predictive utility for intrapartum interventions and early neonatal outcomes, its diagnostic limitations, and its implications for selective versus routine use. Integration of CTG findings with comprehensive clinical assessment is essential to optimize maternal and neonatal outcomes [18].

Objectives of the Review

This systematic review aims to evaluate the clinical utility and predictive reliability of admission CTG in term pregnancies for intrapartum and early neonatal outcomes. The review question was structured using a Population, Intervention/Index test, Comparator, and Outcome (PICO) framework. The population comprised women with term pregnancies at ≥37 weeks of gestation admitted in labor. The intervention or index test was an admission CTG performed at labor ward admission. The comparator included normal/reactive CTG findings, intermittent auscultation, or standard clinical assessment, where reported. The outcomes included intrapartum interventions and early neonatal outcomes.

The primary outcomes were cesarean delivery for suspected fetal distress, instrumental vaginal delivery, and early neonatal compromise, including low Apgar scores and NICU admission. Secondary outcomes included meconium-stained liquor, clinically diagnosed fetal distress, birth asphyxia, neonatal seizures, perinatal mortality, and diagnostic accuracy measures of admission CTG. Diagnostic accuracy was assessed, where data were available, by extracting or reporting sensitivity, specificity, positive predictive value, negative predictive value, and associations between abnormal admission CTG patterns and adverse maternal or neonatal outcomes.

The review also aimed to assess whether routine admission CTG is justified in low-risk term pregnancies or whether a selective, risk-based approach is more appropriate.

## Review

Methodology

To assess the clinical utility of admission CTG in term pregnancies in relation to intrapartum and early neonatal outcomes, a systematic literature review was performed. This review followed a structured approach to ensure comprehensive identification, selection, and synthesis of relevant evidence. The literature search covered studies published from 2000 to 2026, thereby including earlier studies that directly evaluated admission CTG and reported relevant intrapartum or early neonatal outcomes. A total of 252 records were initially identified, of which 211 remained after removal of duplicates. Following screening and eligibility assessment, nine primary studies met the inclusion criteria and were included in the final qualitative synthesis.

The inclusion of nine primary studies reflects the specific eligibility criteria applied in this review rather than an intentionally limited evidence base. Articles were excluded when they did not specifically assess admission CTG, lacked term-pregnancy data, focused only on preterm or non-comparable high-risk groups, did not report intrapartum or early neonatal outcomes, or provided insufficient methodological or outcome data. Multiple databases and reference-list searches were used to improve comprehensiveness; nevertheless, the possibility of missing additional relevant studies remains a limitation and has been acknowledged.

The review was reported in accordance with the Preferred Reporting Items for Systematic Reviews and Meta-Analyses (PRISMA) 2020 reporting framework, where applicable. A formal PRISMA checklist was used to guide reporting completeness; however, the review protocol was not prospectively registered, which is acknowledged as a methodological limitation.

Search Strategy

The search of electronic databases (PubMed, Scopus, Cochrane Library, and Web of Science) was performed for studies published between 2000 and 2026 to identify articles related to the topic. This time frame was selected to capture both contemporary and earlier high-quality evidence on admission CTG, while allowing assessment of changes in obstetric practice, fetal monitoring interpretation, and neonatal outcome reporting over time. The start year of 2000 was chosen to avoid excluding relevant earlier studies that directly evaluated admission CTG in labor ward admission settings. Predefined terms that were searched included admission CTG, admission CTG, term pregnancy, intrapartum outcomes, and neonatal outcomes, with the use of Boolean operators.

These keywords were combined using Boolean operators such as “AND” and “OR” (e.g., “admission CTG AND term pregnancy,” “cardiotocography OR fetal monitoring”) to refine the search and improve retrieval of relevant studies. The core search strategy combined terms for the index test, population, and outcomes. A representative search string was: (“admission cardiotocography” OR “admission CTG” OR “cardiotocography” OR “fetal monitoring”) AND (“term pregnancy” OR “term labour” OR “labour admission”) AND (“intrapartum outcomes” OR “cesarean delivery” OR “instrumental delivery” OR “neonatal outcomes” OR “Apgar score” OR “NICU admission”). This search string was adapted according to the syntax and indexing structure of each database. Only full-text articles published in English were considered. The English-language restriction was applied because of feasibility constraints related to translation and full-text interpretation; however, this restriction may have introduced language bias and is acknowledged as a limitation of the review. The reference lists of the selected studies were also reviewed manually to make sure that any other potentially eligible articles that could have been overlooked during the initial database search were included. Gray literature, unpublished studies, trial registries, and guideline documents from professional organizations were not systematically searched. The review focused on peer-reviewed full-text articles indexed in major scientific databases, supplemented by manual screening of reference lists from selected articles.

Eligibility Criteria

Inclusion criteria: Based on preset inclusion and exclusion criteria, studies were chosen. Inclusion criteria included studies involving term pregnancies, defined operationally as pregnancies at ≥37 completed weeks of gestation, that assessed admission CTG and reported intrapartum or early neonatal outcomes. Admission CTG was defined as a fetal heart rate and uterine contraction tracing performed at the time of admission to the labor ward, before initiation of continuous intrapartum monitoring or before major labor-management decisions were made. Where reported, admission CTG was generally based on an approximately 20-minute recording and classified as reactive/reassuring, suspicious/non-reassuring, or pathological/abnormal according to the criteria used in each study.

Studies including both low-risk and high-risk term pregnancies were eligible if the population consisted of term pregnancies and the outcomes relevant to admission CTG could be extracted. High-risk studies were not excluded outright because admission CTG is frequently used as a triage tool in such populations, and excluding them would have removed clinically relevant evidence on predictive utility. However, risk status was considered during qualitative synthesis, and findings from high-risk populations were interpreted separately from, or with caution in relation to, low-risk term pregnancies.

Eligible study designs included randomized controlled trials, cohort studies, cross-sectional studies, and other observational studies. These heterogeneous study designs were combined because the available evidence on admission CTG is limited and includes both interventional and non-interventional designs. A quantitative meta-analysis was not performed because of methodological and clinical heterogeneity across study designs, risk profiles, CTG classification systems, and outcome definitions. Instead, a qualitative synthesis was used to compare patterns of association, diagnostic performance, and clinical implications across studies. Only peer-reviewed original research articles with clearly defined outcome measures were included to ensure methodological robustness. Peer-reviewed articles in their entirety and published in English only were selected.

Exclusion Criteria

Studies were excluded if they focused exclusively on preterm pregnancies, did not report term-pregnancy data separately, evaluated only continuous intrapartum CTG without a distinct admission CTG assessment, or included high-risk populations without extractable admission CTG-related outcomes. Reviews, editorials, case reports, conference abstracts, non-English articles, and studies with incomplete data, unclear methodology, or irrelevant outcomes were also excluded.

Study Selection

Titles and abstracts were screened first, followed by a full-text assessment of potentially eligible articles. Study selection was performed independently by two reviewers using the predefined eligibility criteria. Disagreements regarding eligibility were resolved through discussion and consensus; where necessary, a third reviewer was consulted. Duplicate records were removed before title and abstract screening, and reasons for full-text exclusion were recorded for PRISMA reporting.

Data Extraction

A standardized data extraction form was used to extract data from the included studies in a consistent manner. Variables extracted comprised author name, year of publication, study design, sample size, population characteristics, classification of CTG findings, and reported intrapartum and neonatal outcomes, including mode of delivery, fetal distress, Apgar scores, and neonatal intensive care unit admissions. Additional extracted variables included country or study setting where available, pregnancy risk status, gestational age criteria, admission CTG definition, CTG classification system, comparator group, and diagnostic accuracy measures, including sensitivity, specificity, positive predictive value, and negative predictive value, where reported.

Data extraction was performed independently by two reviewers. Any disagreement regarding extracted variables or outcome classification was resolved through discussion and consensus; where required, a third reviewer was consulted. Inter-reviewer agreement was not formally quantified using Cohen’s kappa statistic, which is acknowledged as a methodological limitation.

Data extraction was managed using Microsoft Excel (Microsoft Corporation, Redmond, WA, USA; version 365, 2023).

Quality Assessment

Validated appraisal tools suitable for each study design were used to assess methodological quality. Randomized controlled trials were appraised using the Cochrane Risk of Bias 2.0 (RoB 2.0) tool [13], whereas observational studies were appraised using the Newcastle-Ottawa Scale (NOS) [14]. Only primary studies were included in the quality assessment. Review articles and meta-analyses were excluded during full-text eligibility assessment and were not included in the qualitative synthesis or risk-of-bias table.

Risk-of-bias appraisal was performed at the domain level. For randomized studies, RoB 2.0 domains included the randomization process, deviations from intended interventions, missing outcome data, outcome measurement, and selection of reported results. For observational studies, NOS domains included participant selection, exposure ascertainment, cohort comparability, outcome assessment, and adequacy of follow-up. Particular attention was given to CTG-specific methodological concerns, including inconsistent interpretation criteria, lack of blinding, subjective diagnosis of fetal distress, and variation in thresholds for cesarean or instrumental delivery.

Risk-of-Bias Assessment

All included primary studies were assessed for possible sources of bias, including selection bias, performance bias, detection bias, reporting bias, and confounding. The presence and intensity of these factors were used to classify each study as having low, moderate, or high overall risk of bias. Studies were classified as having a moderate risk of bias when limitations were present in one or more domains, such as absence of blinding, incomplete outcome reporting, variability in CTG interpretation, or limited adjustment for confounding, without meeting criteria for high risk across multiple domains.

Overall risk-of-bias ratings were assigned after considering domain-level judgements rather than relying on a single global impression. The assessment also considered whether clinician awareness of admission CTG findings could have influenced subsequent management decisions, particularly cesarean delivery, instrumental delivery, or diagnosis of fetal distress. The qualitative synthesis incorporated these risk-of-bias judgements when interpreting the reliability and applicability of study findings. Study-level risk-of-bias judgements and brief domain-specific justifications are presented in Table 3. Certainty of evidence was not formally assessed using the Grading of Recommendations Assessment, Development and Evaluation (GRADE) approach, which is acknowledged as a limitation.

Data Synthesis

A structured narrative synthesis approach was used because quantitative meta-analysis was not appropriate due to substantial methodological and clinical heterogeneity across study designs, population risk profiles, comparator groups, CTG classification systems, and outcome definitions. Meta-analysis was also limited by incomplete and inconsistent reporting of effect estimates, denominators, confidence intervals, and diagnostic accuracy measures across studies. Therefore, pooled effect estimates, forest plots, meta-regression, and I²-based heterogeneity statistics were not generated. Extracted findings were organized by study design, pregnancy risk status, CTG category, intrapartum outcomes, early neonatal outcomes, and diagnostic accuracy measures.

The synthesis compared outcomes between normal/reactive and suspicious/pathological CTG findings, where reported. Where available, reported odds ratios, p-values, sensitivity, specificity, positive predictive value, negative predictive value, and other effect measures were extracted and summarized descriptively. Because of heterogeneity in population risk profile, subgroup interpretation was undertaken descriptively for low-risk, mixed-risk, and high-risk term pregnancy populations rather than through statistical subgroup analysis. Formal subgroup meta-analysis was not performed because the included studies used inconsistent CTG classification criteria, variable outcome definitions, and heterogeneous study designs. Sensitivity analysis and publication-bias assessment were not performed because the included studies were few in number, heterogeneous in design, and not statistically pooled.

The qualitative synthesis focused on identifying the direction and consistency of associations across studies, particularly the relationship between abnormal admission CTG and cesarean delivery, instrumental delivery, fetal distress, low Apgar scores, neonatal intensive care unit admission, and reported diagnostic accuracy indices. Differences in clinical setting, risk status, interpretation criteria, reported precision measures, and risk of bias were considered when evaluating the strength and applicability of the findings. Risk-of-bias evaluation was conducted using Review Manager (RevMan) software, version 5.4 (The Cochrane Collaboration, 2020). The synthesis should therefore be interpreted as a structured qualitative summary of study-level findings rather than as a pooled statistical estimate of treatment effect or diagnostic accuracy.

Results

Study Selection

**Figure 1:**
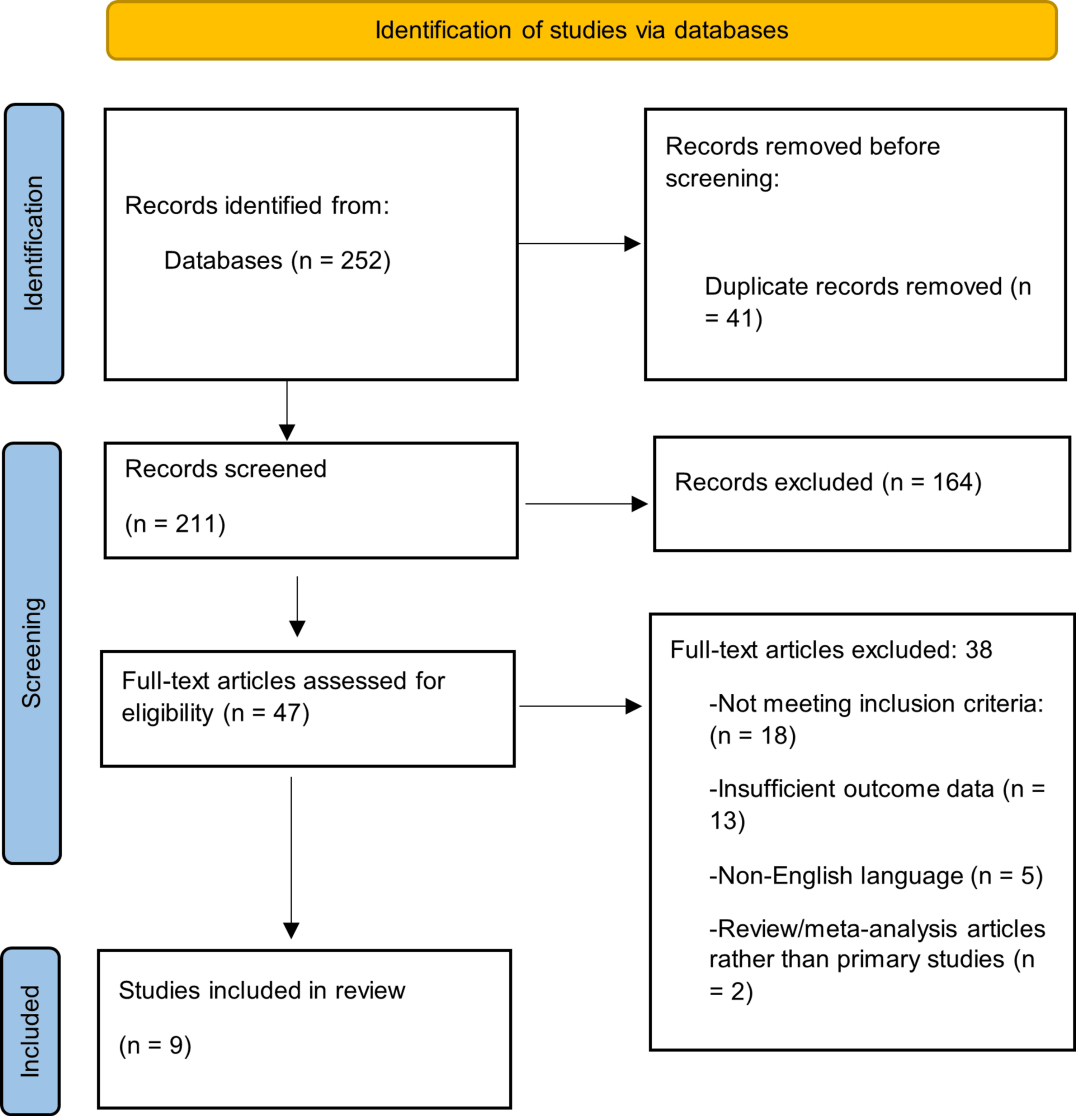
PRISMA flow chart Template derived from the PRISMA 2020 flow chart

A total of 252 records were identified through the initial database search. After the removal of 41 duplicates, 211 unique records were screened. Following title and abstract screening, 164 records were excluded because they were not relevant to the review objective. Therefore, 47 full-text articles were assessed for eligibility. On full-text review, 38 articles were excluded because they did not fulfil the inclusion criteria (n=18), had insufficient outcome data (n=13), were non-English publications (n=5), or were review/meta-analyses articles rather than primary studies (n=2). This selection process resulted in a final sample of nine primary studies included in the qualitative synthesis. The study selection process, including identification, screening, eligibility, and inclusion stages, is presented in the PRISMA flow diagram (Figure 1).

Study Characteristics

The number of participants in the included primary studies varied between 100 and 27,927. The largest study was a retrospective cohort involving 27,927 participants and used computerized analysis of CTG findings. Other studies were smaller prospective or cross-sectional studies, generally involving 100 to 500 participants. All included studies focused on term gestation, defined as ≥37 completed weeks, and evaluated admission CTG performed at labor ward admission. Where reported, admission CTG was based on approximately 20-minute recordings and classified as reactive/reassuring, suspicious/non-reassuring, or pathological/abnormal according to each study’s criteria. Table 1 summarizes the characteristics of the included primary studies, including study design, population size, outcomes assessed, and key findings related to intrapartum interventions and early neonatal outcomes.

**Table 1:** Characteristics and key findings of primary studies on admission cardiotocography and neonatal outcomes CTG: cardiotocography; NICU: neonatal intensive care unit

Study	Admission CTG focus	Study design and population	Main outcomes assessed	Key findings
Bhartiya et al. [16]	Admission CTG in mixed-risk pregnancies	Prospective longitudinal study; n=200	Meconium staining, Apgar score, NICU admission	Abnormal admission CTG was associated with meconium-stained liquor, but predictive value for neonatal outcomes was limited.
Kumar et al. [17]	Admission CTG in term women	Prospective observational study; n=100	Mode of delivery, Apgar score, NICU admission	Abnormal CTG findings were associated with higher cesarean delivery rates and adverse early neonatal outcomes.
Lovers et al. [18]	Computerized CTG analysis in early term labor	Retrospective cohort study; n=27,927	Severe neonatal compromise	Computerized abnormal CTG patterns were associated with increased risk of severe neonatal compromise.
Singh et al. [19]	Intrapartum CTG monitoring	Cross-sectional study; n=500	Birth asphyxia, NICU admission, Apgar score	Non-reassuring CTG findings were associated with increased operative delivery and neonatal complications.
Rahman et al. [20]	Admission CTG in high-risk term pregnancies	Prospective observational study; n=160	Fetal distress, NICU admission	Ominous CTG findings were strongly associated with fetal distress and increased neonatal morbidity.
Akhavan et al. [21]	Admission CTG test	Cross-sectional study; n=425	Cesarean delivery, NICU admission, Apgar score	Admission CTG predicted cesarean delivery and NICU admission, but not neonatal mortality.
Sandhu et al. [22]	Admission CTG screening in high-risk pregnancies	Prospective study; n=150	Fetal distress, NICU admission, Apgar score	Admission CTG showed high specificity and was strongly associated with fetal distress.
Rahman et al. [23]	Admission CTG in a low-resource setting	Observational study	Perinatal outcomes	Admission CTG was useful for triaging cases at increased risk of adverse intrapartum and neonatal outcomes.
Nazir et al. [24]	Admission CTG and Apgar score correlation	Observational cross-sectional study	Prediction of low Apgar score	Admission CTG showed moderate sensitivity and high specificity for predicting low Apgar score.

Characteristics of Admission CTG Findings

**Figure 2:**
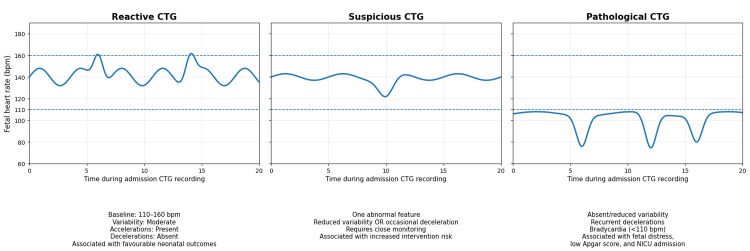
Schematic representation of admission CTG patterns and associated intrapartum and early neonatal outcomes Created by authors using Python and Matplotlib in Google Colab without the use of generative artificial intelligence tools. This figure illustrates the characteristic features of reactive, suspicious, and pathological cardiotocography (CTG) patterns based on fetal heart rate baseline, variability, accelerations, and decelerations. Reactive patterns demonstrate a normal baseline with moderate variability and accelerations, suspicious patterns show reduced variability or occasional decelerations, and pathological patterns exhibit absent/reduced variability with recurrent decelerations or bradycardia. The associated clinical outcomes depicted are derived from the qualitative synthesis of included studies, including increased risk of obstetric intervention, fetal distress, low Apgar scores, and neonatal intensive care unit (NICU) admission. This figure is a conceptual and descriptive schematic based on standard clinical definitions and the qualitative synthesis. It does not represent individual patient data, pooled quantitative analysis, or meta-analytic effect estimates.

In general, most individuals exhibited reactive and normal CTGs, while others exhibited suspicious or pathological CTGs. However, the distribution of CTG patterns varied substantially across studies, likely reflecting differences in study populations and clinical settings. For instance, in a study that involved the largest cross-section of the population, 39.36% had reactive CTG, whereas 54.68% had pathological or non-reactive CTGs. Likewise, in another study, 92% were found to exhibit normal CTGs, 7% non-reassuring CTGs, and 1% abnormal CTGs. Figure 2 shows a schematic representation of CTG patterns and their associated clinical outcomes.

Intrapartum Outcomes

There is a consistent relationship between abnormal or non-reactive CTG results and increased intervention in obstetrics. Cesarean sections and operative deliveries were more frequent among patients who had suspicious and pathological results on their CTGs. In one particular study, 47.77% of women underwent cesarean delivery in the presence of suspicious CTG findings. Operative deliveries among patients with non-reassuring CTGs also significantly increased (p < 0.0001). Among high-risk patients who had abnormal CTGs, there was a significantly higher proportion with fetal distress (73%) compared to those with normal CTGs (15%). These findings suggest an association between abnormal admission CTG and intrapartum intervention; however, causality cannot be inferred because most included studies were observational, and clinical management may have been influenced by awareness of CTG findings.

Early Neonatal Outcomes

There was a significant association between neonatal outcomes and CTG findings. It was observed that the incidence of Apgar scores less than 8 increased in the presence of pathological CTG patterns. One study reported that 63.40% of neonates with pathological CTG had Apgar scores below 8. This relationship was further supported by another study in which pathological CTG was associated with a reduced likelihood of higher Apgar scores (OR 0.30, p<0.001). NICU admission rates were also higher among the abnormal CTG group, reaching 32.34% in one study.

Moreover, a significant association was observed between adverse neonatal conditions and CTG patterns. These included seizures, birth asphyxia, and increased risk of mortality. Although overall mortality rates were low, they were more frequently observed in cases with ominous CTG patterns compared to reactive CTG. Therefore, although abnormal admission CTG was associated with adverse early neonatal outcomes, the predictive value remained inconsistent across outcomes and study populations.

Diagnostic Value of Admission CTG

The admission CTG showed varying performance levels across studies. To improve interpretability, diagnostic performance was summarized according to the outcome assessed, because sensitivity, specificity, positive predictive value, and negative predictive value differed across low Apgar score, birth asphyxia, fetal distress, and neonatal intensive care unit admission. The sensitivity was generally moderate, while the specificity was consistently higher in several studies. This is reflected in findings where the sensitivity and specificity for predicting low Apgar scores were 66.7% and 93.3%, respectively. In another study, specificity and negative predictive values were high; however, sensitivity and positive predictive values were poor for the diagnosis of birth asphyxia, indicating variability in predictive performance depending on the outcome measured. Admission CTG appeared more useful for ruling out adverse outcomes when findings were reactive or reassuring than for definitively predicting adverse outcomes when findings were suspicious or pathological. This pattern supports its role as a screening tool rather than a standalone diagnostic test. The diagnostic performance of admission CTG across reported outcomes is summarized in Table 2.

**Table 2:** Summary of diagnostic performance of admission CTG across reported outcomes Diagnostic performance could not be pooled because of heterogeneity in CTG classification criteria, population risk status, and outcome definitions. CTG: cardiotocography; NICU: neonatal intensive care unit

Outcome assessed	Diagnostic performance reported	Interpretation
Low Apgar score	Sensitivity 66.7%; specificity 93.3%	Moderate sensitivity with high specificity, suggesting better ability to identify neonates unlikely to have low Apgar scores when CTG is reassuring.
Birth asphyxia	High specificity and negative predictive value; poor sensitivity and positive predictive value	Reactive CTG may help exclude birth asphyxia, but abnormal CTG alone has limited ability to confirm it.
Fetal distress	Abnormal CTG associated with higher fetal distress rates	Association present, but diagnostic accuracy varied because fetal distress definitions and intervention thresholds differed across studies.
NICU admission	Higher NICU admission rates among abnormal CTG groups	Abnormal CTG was associated with increased NICU admission, but predictive performance was inconsistent across populations.

Quality Assessment Findings

There was variation in the methodological quality of the included studies, with most observational studies demonstrating moderate to good methodological quality. Cohort studies involving large sample sizes showed stronger methodological rigor due to better statistical power and structured data collection, whereas smaller studies were more prone to sampling limitations and potential confounding factors.

Risk-of-Bias Across Studies

Risk-of-bias appraisal demonstrated heterogeneity across the included studies, with identified risks including selection bias, lack of blinding, variability in CTG interpretation, and inconsistent clinical management protocols. Subjective interpretation of CTG findings and clinician awareness of admission CTG results were important sources of potential performance and detection bias, particularly because CTG findings may influence decisions regarding cesarean or instrumental delivery. As depicted in Table 3, most studies were classified as having a moderate overall risk of bias based on domain-level limitations rather than pervasive high-risk features across all domains.

**Table 3:** Risk-of-bias assessment of included primary studies

Study (Reference)	Study design	Selection bias	Performance bias	Detection bias	Overall risk of bias	Brief justification
Bhartiya et al. [16]	Prospective longitudinal	Moderate	Moderate	Moderate	Moderate	Selection criteria and risk status were not fully standardised; no blinding of CTG-based management.
Kumar et al. [17]	Prospective observational	Moderate	Moderate	Moderate	Moderate	Small observational study with possible selection bias and non-blinded intervention decisions.
Lovers et al. [18]	Retrospective cohort	Low	Moderate	Moderate	Moderate	Large cohort and computerised CTG reduced selection bias, but retrospective design limited control over management variation.
Singh et al. [19]	Cross-sectional	Moderate	Moderate	Moderate	Moderate	Cross-sectional design limited causal inference; CTG knowledge may have influenced delivery decisions.
Rahman et al. [20]	Prospective observational	Moderate	Moderate	Moderate	Moderate	High-risk population limited generalisability; non-blinded CTG interpretation may have influenced outcomes.
Akhavan et al. [21]	Cross-sectional	Moderate	Moderate	Moderate	Moderate	Potential selection bias and non-blinded CTG interpretation limited internal validity.
Sandhu et al. [22]	Prospective study	Moderate	Moderate	Moderate	Moderate	High-risk cohort and lack of blinding reduced applicability to general term pregnancies.
Rahman et al. [23]	Observational	Moderate	Moderate	Moderate	Moderate	Variable monitoring protocols and non-blinded outcome assessment increased bias risk.
Nazir et al. [24]	Cross-sectional	Moderate	Moderate	Moderate	Moderate	Limited confounder control and cross-sectional design resulted in moderate bias.

Narrative Synthesis of Findings

Narrative synthesis showed three main patterns across the included primary studies. First, abnormal or non-reactive admission CTG findings were consistently associated with higher rates of intrapartum intervention, particularly cesarean delivery and instrumental delivery. Second, abnormal CTG patterns were associated with adverse early neonatal outcomes, including low Apgar scores and NICU admission, but these associations were not consistent enough to establish admission CTG as a definitive diagnostic test. Third, diagnostic performance varied by outcome, with admission CTG generally showing higher specificity and negative predictive value than sensitivity and positive predictive value. Because the included studies differed in design, population risk status, CTG classification criteria, outcome definitions, and statistical reporting, these findings were interpreted descriptively rather than pooled quantitatively.

**Figure 3:**
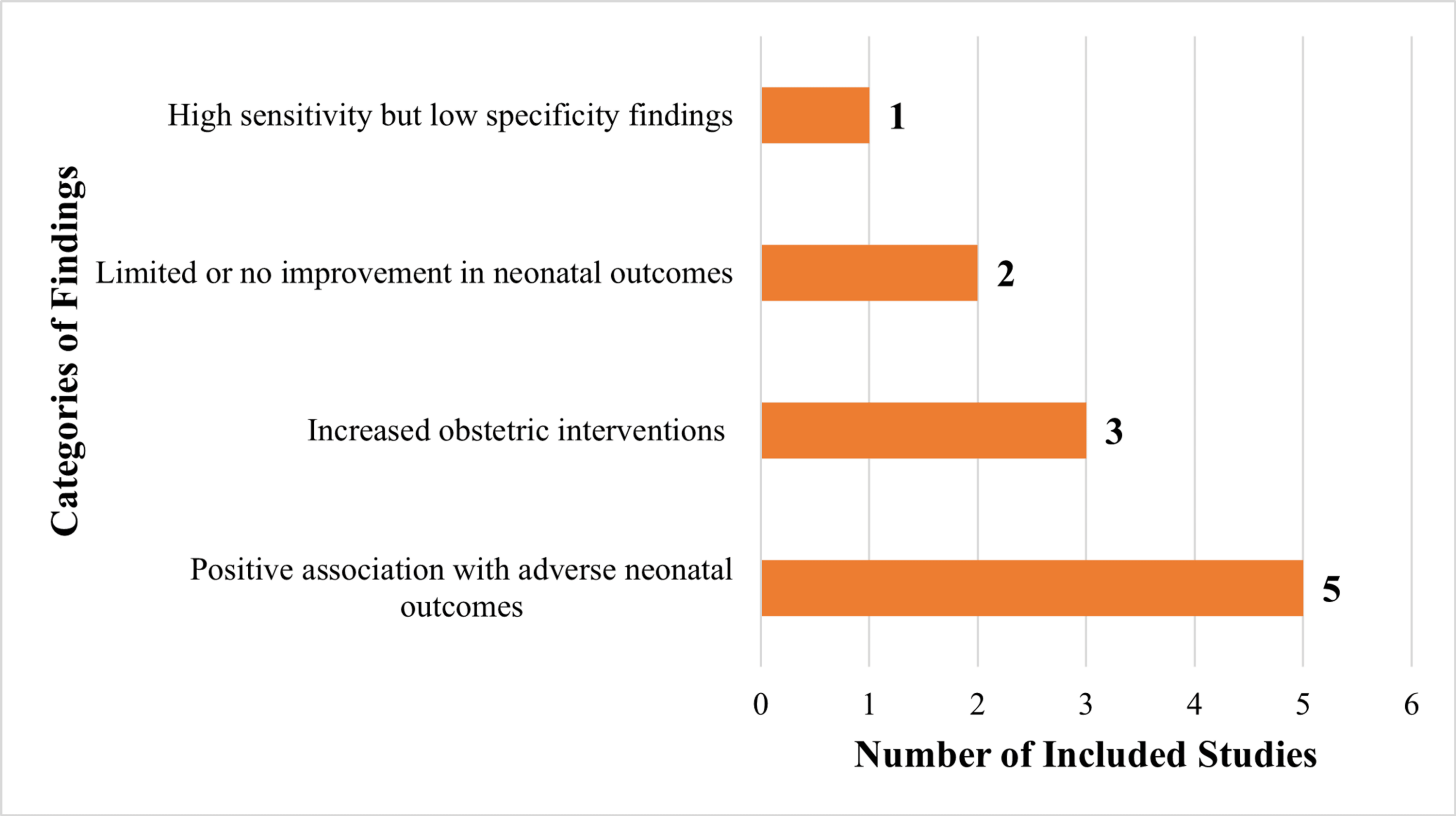
Distribution of synthesized findings across included primary studies This figure provides a descriptive summary of the distribution of findings reported across the included primary studies. It is intended to illustrate study-level patterns observed in the narrative synthesis and should not be interpreted as pooled quantitative evidence, meta-analysis, or a weighted estimate of effect.

Taken together, these findings indicate that admission CTG may function as a screening tool for identifying term pregnancies that require closer surveillance, particularly when combined with clinical risk assessment. However, its routine use in low-risk term pregnancies may increase obstetric intervention without consistent evidence of proportional neonatal benefit. The synthesis should therefore be interpreted as a structured qualitative summary of study-level findings rather than as a pooled statistical estimate of treatment effect or diagnostic accuracy. Figure 3 illustrates the synthesized distribution of findings across the included primary studies.

As with Figure 2, Figure 3 should be interpreted as a descriptive summary of study-level findings rather than a pooled quantitative analysis.

Discussion

A systematic review was conducted in the present study to evaluate the usefulness of admission CTG in term pregnancies for predicting intrapartum and early neonatal outcomes. The findings demonstrate that CTG at admission is associated with increased detection of fetal compromise and a higher likelihood of obstetric interventions, including cesarean and instrumental deliveries. However, improvements in key neonatal outcomes, such as Apgar scores and NICU admissions, were not consistently observed. These findings are consistent with large Cochrane reviews indicating that although CTG enhances the detection of fetal distress, it does not significantly reduce perinatal mortality or long-term neonatal morbidity [1]. This suggests that while CTG is useful as a surveillance tool, its routine use in low-risk pregnancies may not be justified.

The diagnostic performance of admission CTG demonstrated variability across studies. While sensitivity was generally moderate, specificity was relatively higher in certain studies, particularly in predicting adverse neonatal outcomes such as low Apgar scores. Despite this, the overall predictive accuracy remained inconsistent. This apparent discrepancy may be explained by the influence of clinical interpretation and decision-making, where cautious interpretation of non-reassuring tracings may lead to increased intervention rates. Consequently, elevated rates of obstetric interventions may reflect clinical practice patterns rather than the intrinsic diagnostic limitations of CTG alone.

Current literature indicates that CTG contributes to increased rates of cesarean and instrumental deliveries without a proportional improvement in neonatal outcomes [1]. Comparative analyses with adjunctive techniques, such as fetal electrocardiogram (ECG) ST-segment waveform analysis, have yielded inconsistent findings, with no clear evidence of superiority in improving neonatal health outcomes [5]. Furthermore, network meta-analyses support the observation that intensified fetal surveillance strategies may increase intervention rates without corresponding neonatal benefit [8].

Variability in CTG interpretation remains a significant limitation affecting its clinical utility. Substantial interobserver and intraobserver differences have been documented, leading to inconsistent clinical decisions based on similar CTG tracings [10]. This subjectivity reduces the reliability of CTG as a standalone diagnostic modality and underscores the importance of standardized interpretation protocols [25]. Although artificial intelligence-based systems have been explored to reduce interpretative variability, their application to routine admission CTG remains insufficiently validated and should not replace structured clinical assessment.

Physiological complexity also limits the predictive precision of CTG. Fetal heart rate variability reflects interactions between autonomic nervous system activity and cardiovascular regulation, making it difficult to distinguish adaptive fetal responses from pathological compromise using CTG alone [6]. These factors contribute to limitations in predictive precision despite observable associations with adverse outcomes.

Comparison with alternative monitoring approaches provides additional context. Intermittent auscultation has demonstrated similar neonatal outcomes with fewer obstetric interventions in low-risk pregnancies [11]. In addition, broader evaluations of intrapartum fetal surveillance have not identified a clearly superior monitoring strategy for improving maternal and neonatal outcomes [8]. These findings support a selective, risk-based approach to CTG use rather than routine implementation in all term pregnancies.

Discussion of advanced digital systems and machine learning was limited to contextual relevance because this review focused on admission CTG rather than validation of emerging monitoring technologies. Future research may evaluate whether such tools can improve standardisation, but current evidence remains insufficient to support their routine use for admission CTG decision-making.

A central implication of the synthesis is that admission CTG should be interpreted as a risk-stratification tool rather than as a definitive diagnostic test. Across the included primary studies, abnormal or non-reactive CTG findings were associated with higher intervention rates and some adverse neonatal outcomes; however, heterogeneity in population risk status, CTG classification criteria, outcome definitions, and clinical management protocols limits the strength of these associations. This is particularly important in low-risk term pregnancies, where unnecessary escalation of care may occur when equivocal CTG findings are interpreted without sufficient clinical context.

An important consideration in CTG use is the balance between early detection of fetal compromise and the risk of overtreatment. Increased intervention rates associated with CTG may lead to unnecessary surgical and instrumental deliveries, which can contribute to maternal morbidity and increased healthcare resource utilization without clear neonatal benefit [1,8]. This highlights the need for cautious interpretation of CTG findings within the broader clinical context.

The findings of this review indicate that while admission CTG is effective in identifying fetuses at potential risk, its predictive value for neonatal outcomes remains limited. Associations between abnormal CTG patterns and adverse neonatal outcomes were observed; however, these associations do not consistently translate into improved clinical outcomes when CTG is used as a routine screening tool. Therefore, CTG should be interpreted in conjunction with comprehensive clinical assessment and other diagnostic modalities. Future research should focus on refining risk-based protocols, improving standardization of interpretation, and evaluating the integration of emerging technologies only after adequate validation in admission CTG populations.

Limitations and Future Directions

The limitations of this review include heterogeneity in study design, sample size, and outcome measures, which precluded quantitative meta-analysis. The majority of selected studies had observational designs, resulting in moderate risks of bias and limited ability to infer causation. Variations in CTG interpretation criteria across studies were additional sources of inconsistency. Differences in population risk status, including low-risk, mixed-risk, and high-risk term pregnancies, also limited direct comparability across studies. No pooled effect estimates, forest plots, formal heterogeneity statistics, sensitivity analyses, or publication-bias assessments were generated because the included studies were few in number, clinically heterogeneous, and inconsistently reported effect estimates, denominators, confidence intervals, and diagnostic accuracy thresholds. This limits statistical precision and prevents firm conclusions regarding the magnitude of association between admission CTG findings and clinical outcomes. The prevalence of single-center studies and the lack of diverse samples may limit the generalizability of the evidence. The restriction to English-language full-text articles may have introduced a language bias. Inter-reviewer agreement was not formally quantified using Cohen’s kappa statistic, which is another methodological limitation. The search strategy did not include gray literature, unpublished studies, trial registries, or a systematic search of professional guidelines from major obstetric organizations; therefore, some relevant non-indexed or guideline-based evidence may have been missed. The review protocol was not prospectively registered, including in PROSPERO, and the certainty of evidence was not formally assessed using the Grading of Recommendations Assessment, Development and Evaluation (GRADE) approach. Publication bias could not be formally assessed because a meta-analysis was not performed. These limitations may reduce reproducibility and restrict the strength of conclusions drawn from the qualitative synthesis.

Future research should include large-scale multicenter randomized controlled trials to develop standardized protocols for the use of admission CTG. The integration of artificial intelligence and sophisticated technology may help improve CTG interpretation, but its clinical utility in admission CTG requires further validation. There is a need for more research comparing selective versus routine use of admission CTG in low-risk term pregnancies. Future studies should also use standardized CTG classification criteria, predefined neonatal outcome measures, and stratified analyses by pregnancy risk status to improve comparability and clinical applicability.

## Conclusions

Admission cardiotocography continues to be widely used as a method of intrapartum fetal surveillance in term pregnancies. Its clinical utility lies in continuous monitoring of fetal heart rate patterns and uterine contractions, allowing early identification of potential fetal compromise. Based on the findings of this review, admission CTG is associated with increased detection of fetal distress and a higher likelihood of obstetric interventions, including cesarean and instrumental deliveries. However, its predictive value for adverse neonatal outcomes remains inconsistent, reflecting variability in sensitivity and specificity across studies as well as the influence of clinical interpretation on intervention decisions. Although abnormal CTG patterns are associated with outcomes such as low Apgar scores and increased NICU admissions, these associations do not consistently translate into improved neonatal outcomes. Therefore, routine use of admission CTG, particularly in low-risk pregnancies, may lead to increased intervention rates without proportional clinical benefit. A selective, risk-based approach with integration of clinical assessment is recommended to optimize maternal and neonatal outcomes.
